# Evaluating the Feasibility and Acceptability of Internet-Based Cognitive Behavioral Therapy for Insomnia in Rural Women

**DOI:** 10.1089/whr.2020.0053

**Published:** 2020-05-05

**Authors:** Mairead Eastin Moloney, Madeline Dunfee, Matthew Rutledge, Nancy Schoenberg

**Affiliations:** ^1^ Department of Sociology, College of Arts and Sciences, University of Kentucky, Lexington, Kentucky, USA.; ^2^ Department of Behavioral Science, College of Medicine, University of Kentucky, Lexington, Kentucky, USA.; ^3^ Department of Statistics, College of Arts and Sciences, University of Kentucky, Lexington, Kentucky, USA.

**Keywords:** acceptability, brief interventions, feasibility, insomnia, technology

## Abstract

***Background:*** Insomnia, one of the most common sleep disorders among women in midlife, is associated with multiple negative health outcomes. Rural Appalachian women are disproportionately affected by insufficient sleep, but their barriers to care (*e.g.*, health care shortages, cultural norms) may prevent intervention. This study assessed the feasibility and acceptability of Sleep Healthy Using the Internet (SHUTi) an Internet-based version of cognitive behavioral therapy for insomnia in Appalachian women ages 45+ years.

***Materials and Methods:*** We used mixed methods to assess feasibility (through summaries of recruitment and retention data) and acceptability (quantitatively through online survey scales and qualitatively through interviews). Subject-level responses for satisfaction, adherence, and helpfulness scales were averaged over the multiple response domains and reported as percentages. Interviews were transcribed and coded using a multistage coding process.

***Results:*** Forty-six women (average age 55 years) enrolled; 38 completed the SHUTi program (retention = 82.6%). The majority of participants (61%) indicated that SHUTi made things “somewhat better” or “a lot better.” Seventy-six percent reported that they followed the SHUTi protocol “most of the time” or “consistently.” Most participants (84%) ranked SHUTi as “moderately” or “very” helpful. Participants expressed enthusiasm about SHUTi and offered minor suggestions for improvement.

***Conclusions:*** This study was the first to asses SHUTi in the health disparity population of Appalachian women. Rich insights gained through quantitative and qualitative data suggest that SHUTi was feasible and acceptable for middle-aged Appalachian women. Given rural Appalachian women's documented barriers to utilizing technology, these results hold promise for SHUTi's utility in other rural populations. Future research should incorporate a randomized case–control design within a larger sample and consider participants' suggestions for improvement.

## Introduction

Sleep is increasingly recognized as critical to health,^1^ yet disordered sleep, especially among women, is prevalent.^2–4^ About 33%–36% of premenopausal women report insomnia and rates are even higher (44%–61%) in postmenopausal women.^5^ This study answers the call to implement, adapt, and evaluate evidence-based interventions addressing insufficient sleep in women.^6^ Such research is particularly critical for women who, by virtue of their geographic location, race/ethnicity, low socioeconomic status, high disease rates, and/or rural status, are members of health disparity populations.^7–9^ Thus, we focus on women in the Appalachian region of the United States, an area with high rates of poverty, health care shortages, morbidity, and mortality, and target the age group (45+ years) most likely to be diagnosed with and treated for insomnia.^4,5,10,11^

Although insufficient sleep is differentially distributed across the United States, a recent analysis of county-level data found that the central region within Appalachia, specifically 84 counties traversing Eastern Kentucky, Western West Virginia, Northeast Tennessee, Western Virginia, and Southern Ohio, has the highest aggregation of reported insomnia “hotspots” in the nation. In these counties, 25%–58% of adults report insufficient sleep 15+ nights out of 30.^12^ Appalachian women not only live within this hotspot, they possess multiple biopsychosocial factors (*e.g.*, older age, female sex, low socioeconomic status, high depression rates) that place them at heightened insomnia risk.^1,10,13,14^ Although intervention is warranted, regional considerations (*e.g.*, health care shortage, high rates of prescription drug abuse, transportation barriers) suggest a self-administered technologically facilitated insomnia intervention may be ideal for this population.^10,15^

**FIG. 1.**
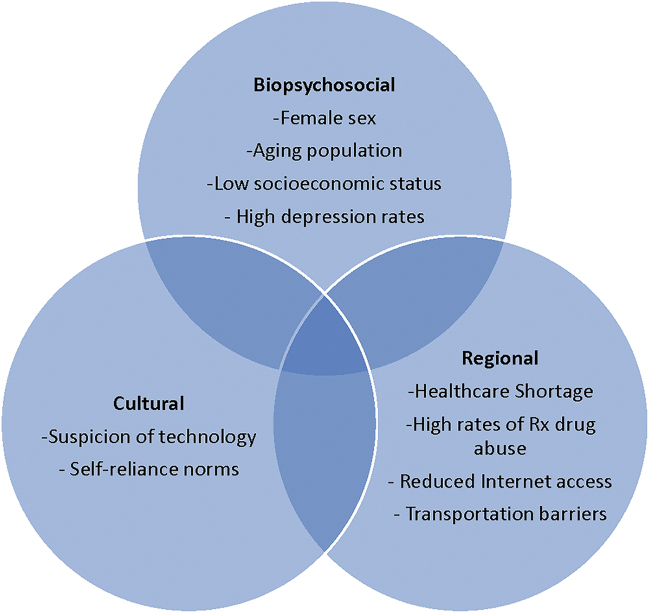
Confluence of biopsychosocial, regional, and cultural factors necessitating an assessment of a technologically facilitated nonpharmacological insomnia intervention in Appalachian women.

To date, however, technology-based interventions are rare in Appalachia and previous research suggests that sociocultural factors shape Appalachian women's treatment preferences and technology use. For instance, Snell-Rood et al. found that help-seeking behaviors among Appalachian women with depression were impeded by strong norms of self-reliance.^14^ A separate study found that Appalachian adults regretted the loss of self-reliance resulting from technology use and generally regarded technology with suspicion.^16^ Given these intersecting factors, a study of the feasibility and acceptability of a technologically facilitated insomnia intervention was necessary (Fig. 1).

We employed “Sleep Healthy Using the Internet” (SHUTi), an Internet-based version of cognitive behavioral therapy for insomnia (CBT-I). SHUTi is the most widely used and well-validated version of Internet-based CBT-I.^17^ Previous studies have found it efficacious for up to 12 months.^18^ SHUTi has demonstrated efficacy in adults with insomnia,^19^ adults with asthma,^20^ and cancer survivors.^21^ Notably, this study is the first to assess SHUTi in a rural health disparity population; the program's cocreators expressed openness to modifying SHUTi based on our participants' feedback (L. Ritterband and F. Thorndike, pers. comm.).

We used both quantitative (survey) and qualitative (semistructured interviews) methods to determine SHUTi's feasibility and acceptability among Appalachian women ages 45+ years. Taking a mixed methods approach allowed for more nuance and depth of data, even within a relatively small sample.^22^ Our findings suggest SHUTi's utility among other populations of rural women. Results related to the present intervention's effectiveness are reported elsewhere.^23^

## Materials and Methods

### Participants

Participants were recruited between January 2018 and April 2018 with the help of the University of Kentucky's Center of Excellence in Rural Health in Appalachian Kentucky. Recruitment techniques included paper and electronic flyers, provider referrals to an informational Facebook page, and snowball sampling. Interested participants were screened over the telephone. Eligibility criteria included (1) being female and 45 years or older, (2) living in Appalachian Kentucky, (3) self-reporting difficulty falling/staying asleep ≥3 nights a week for ≥3 months, (4) using or previously using prescription or over-the-counter sleep aids (*e.g.*, zolpidem, diphenhydramine) ≥3 months, and (5) having regular Internet access. Exclusion criteria included obstructive sleep apnea, schizophrenia, dementia, Alzheimer's disease, Cushing's disease, or bipolar disorder with psychosis. The University of Kentucky's Institutional Review Board approved the study and all participants provided informed consent.

### Procedures

Interested participants (*N* = 68) contacted the study's principal investigator (PI) or study coordinator and were screened for eligibility over the telephone. Eligible participants (*N* = 46) completed an online survey in REDCap and a semistructured in-person or telephone interview with the PI or study coordinator before receiving SHUTi access.

Consistent with the SHUTi program, participants were required to complete the six, once-weekly cores (insomnia overview, sleep restriction, stimulus control, cognitive restructuring, sleep hygiene, and relapse prevention) in 9 weeks.^20^ Cores took an average of 45 minutes to complete. Participants received daily e-mail reminders to complete an 11-item sleep diary.^20,24^ SHUTi completers (*N* = 38) took part in a postintervention survey and interview. For additional information on the SHUTi program.^24^ Participants received $50 gift cards after pre- and postintervention assessments.

### Measures

We report feasibility as summaries of recruitment and retention data. Acceptability was assessed quantitatively through online survey scales measuring SHUTi satisfaction, adherence, and perceived helpfulness. We also explored our outcomes of interest during the qualitative interviews.

**FIG. 2.**
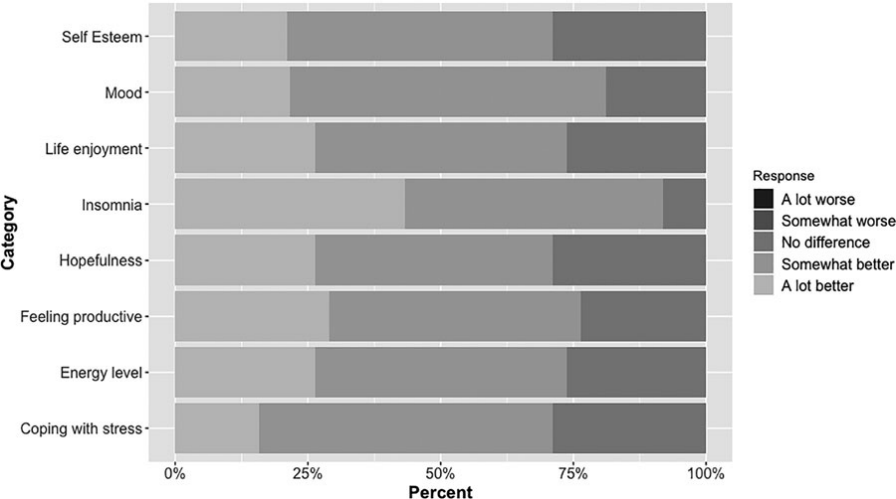
Participant satisfaction.

Consistent with previous CBT-I research assessing participant satisfaction,^25^ we adapted the Consumer Report Treatment satisfaction scale^26^ to ask: “How much do you feel the SHUTi treatment program has helped you in the following areas?” Items (*i.e.*, self-esteem, mood, life enjoyment, insomnia) were rated on a 5-point Likert scale (from “a lot worse” = 1 to “a lot better” = 5) (Fig. 2).

**FIG. 3.**
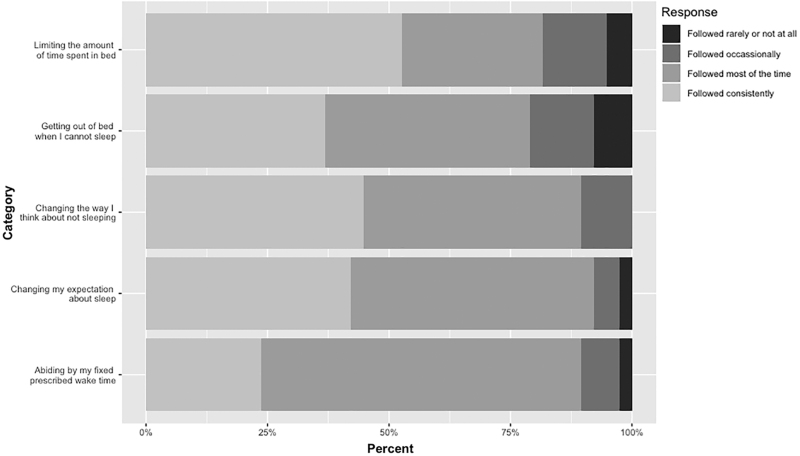
Participant adherence.

Self-report scales of adherence and perceived helpfulness of treatment were adapted from Manber et al.^25^ For adherence we asked: “How closely were you able to follow these SHUTi components?” Using a 4-point Likert scale (0 = “followed rarely or not at all” to 3 = “followed: consistently”), participants assessed their adherence to limiting the amount of time I spend in bed; getting out of bed when I cannot sleep; changing the way I think about not sleeping; changing my expectations about sleep; abiding by my prescribed wake time (Fig. 3).

**FIG. 4.**
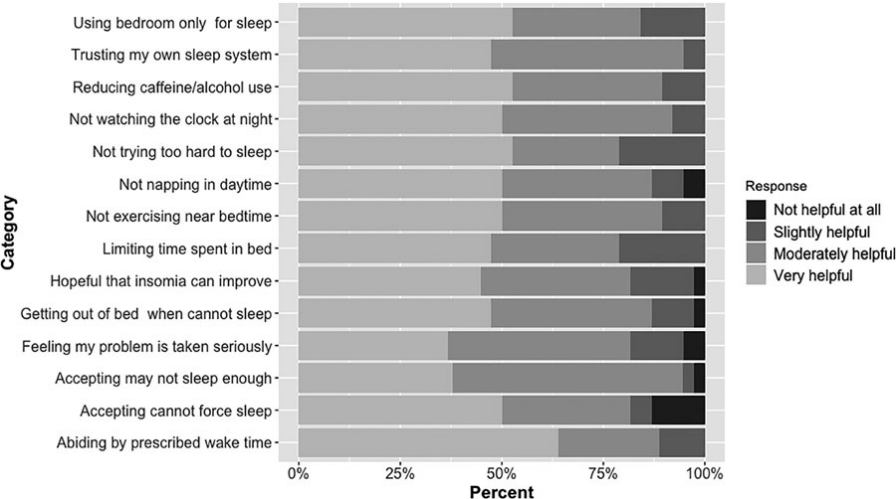
Helpfulness of Sleep Healthy Using the Internet.

To assess helpfulness, we asked: “How helpful were the following components of the SHUTi program?” There were 14 components related to sleep habits and attitudes: using bedroom only for sleep; trusting my own sleep system; reducing caffeine/alcohol use; not watching the clock at night; not trying too hard to sleep; not napping in daytime; not exercising near bedtime; limiting time spent in bed; abiding by prescribed wake time; getting out of bed when cannot sleep; accepting they may not sleep enough; accepting that sleep cannot be forced; feeling my problem is taken seriously; feeling hopeful that insomnia can improve (Fig. 4). Participants used a 4-point Likert scale (0 = “not helpful at all” to 3 = “very helpful.”).

Baseline and postintervention semistructured interviews were completed in-person or over the telephone, depending on what was more convenient for the participant. Interviews were digitally recorded and professionally transcribed. At baseline and postintervention participants were asked to discuss their sleep experiences and any previous sleep remedies they had tried. During the postintervention interviews we asked participants to assess the SHUTi program's acceptability and offer suggestions for improvement.

### Analysis

We employed a single group, pretest, post-test design. Descriptive statistics were calculated for all participants. Subject-level responses for the satisfaction, adherence, and helpfulness scales were averaged over the multiple response domains and reported as percentages.

We used NVivo 12.0 qualitative analytic software^27^ to organize qualitative coding and facilitate analysis. Analyses were informed by grounded theory and we used a multistage inductive coding process. This method allowed us to focus our analyses on sleep experiences and SHUTi, while also remaining open to tangential and emergent themes.^28^

## Results

### Participant characteristics

Thirty-eight women completed the intervention. The average participant age was 55 years; this is older than the average age of women in the region (41.9 years),^29^ but appropriate for our focus on women ages 45+ years. Consistent with the regional demographics, 95% of participants were white non-Hispanic. The majority were married, employed full-time, and had <2 years of college education (Table 1).

**Table 1.** Demographics of Sleep Healthy Using the Internet participants who completed the study (*N* = 38) versus those who did not (*N* = 8)

Characteristics	Noncompleters (n* = 8), *n (%)	Completers (n* = 38), *n (%)	p
Age, mean (SD)	58.1 (7.9)	55.1 (6.2)	0.23
Education			0.17
<HS	1 (12.5)	0 (0)	
HS/GED	2 (25)	10 (26.3)	
Some college	2 (25)	11 (28.9)	
Vocational training	0 (0)	2 (5.3)	
Associates	1 (12.5)	5 (13.1)	
Bachelors	0 (0)	6 (15.8)	
Some graduate/masters	2 (25)	1 (2.6)	
PhD/MD/professional	0 (0)	3 (7.9)	
Ethnicity			0.99
White/Caucasian	8 (100)	36 (94.7)	
Black	0 (0)	1 (2.6)	
American Indian	0 (0)	1 (2.6)	
Private insurance	3 (37.5)	35 (92.1)	**0.002**
Medicare	3 (37.5)	4 (10.5%)	0.09
Medicaid	2 (25)	1 (2.6)	0.07
Marital status			0.16
Married	4 (50)	28 (73.7)	
Living as married	1 (12.5)	2 (5.3)	
Divorced	1 (12.5)	6 (15.8)	
Widowed	1 (12.5)	1 (2.6)	
Separated	1 (12.5)	0 (0.0)	
Single, never married	0 (0)	1 (2.6)	
Employment status			0.068
Self-employed	0 (0)	1 (2.6)	
Full-time	4 (50)	29 (76.3)	
Homemaker	0 (0)	2 (5.3)	
Not working (disability)	3 (37.5)	2 (5.3)	
Retired	1 (12.5)	1 (2.6)	
No response	0	3 (7.9)	
Health status			0.39
Excellent	0 (0)	1 (2.6)	
Very good	5 (62.5)	16 (42.1)	
Good	2 (25)	14 (36.8)	
Fair	1 (12.5)	7 (18.4)	
Any health condition	2 (25)	14 (36.8)	0.69

GED, General Educational Development (High School Equivalency Diploma); HS, High School; SD, standard deviation.

### Feasibility

Forty-six women enrolled and 38 completed the intervention; the retention rate was 82.6%. Twenty-two additional women demonstrated interest in being recruited into the study but did not qualify because they lived outside of Appalachian Kentucky. The analyzed sample (*N* = 38) was similar to the entire sample (*N* = 46), although completers were significantly more likely to have private insurance (92% vs. 38%).

### Acceptability

#### Quantitative results

Participants were generally satisfied with SHUTi. Most participants (61%) thought that SHUTi made things “somewhat better” or “a lot better.” None of the participants thought SHUTi “made things worse.” The mean self-reported satisfaction score was 4.0 out of 5.0 (95% confidence interval [CI]: 3.8–4.2).

Seventy-six percent reported following the SHUTi protocols “most of the time” or “consistently.” On average, participants reported following protocols at least most of the time (*p* < 0.001, *t*-test on subject-level scores averaged over protocols). The mean self-reported adherence score was 2.2 out of 3.0 (95% CI: 2.0–2.4).

Most (84%) participants found SHUTi to be “moderately helpful” or “very helpful” in 14 different areas associated with sleep (Fig. 1). The mean helpfulness score was 2.3 out of 3.0 (95% CI: 2.1–2.5) and on average participants found the SHUTi program to be at least “moderately helpful” with improving sleep (*p* < 0.001).

Participant self-reports of satisfaction, adherence, and helpfulness are verified by participant login data, sleep diary completion, core completion, and the significant postintervention improvements in sleep as measured by the Insomnia Severity Index^30^ (15.1–6.5) and the Pittsburgh Sleep Quality Index^31^ (12.1–8.5).^23^

#### Qualitative results

Participant interviews lasted an average of 22:40 minutes (range: 11:59–46:44), with preintervention interviews generally lasting longer than postintervention interviews. Participants were generally enthusiastic about the SHUTi program. Many participants described it as “a good program”; others noted “it was very helpful, very interesting.” Some participants described it as “very educational.” Participants also discussed the program's user-friendly nature (*e.g.*, “I thought it was very easy to complete actually”) and others described SHUTi as “easy to navigate through.” The flexibility and independent nature of the program also contributed to its acceptability. As one participant explained, “I enjoyed the part where I could do it on my time. I enjoyed that and it was not like you have to go somewhere and meet up with a bunch of people to do it.” Several participants described recommending SHUTi to others (*e.g.*, “I've recommended it to a lot of people”) and mentioned being “thankful” for the program.

When asked how to improve SHUTi's acceptability, several participants suggested extending the sleep diary window beyond 3 days to increase participation among individuals with limited ability to log on to the system. Reasons for inability to log on with greater frequency included: “work,” “leading a busy life,” “travel,” and “being a caretaker.” A few felt the program was generally “too long” or “time-consuming.” Given the limitations of a rural regional context, some participants were only able to complete the SHUTi program on their work computers, as they lacked home computers or smartphones. As one participant explained: “[W]hen you're at work if you don't have that 45 minutes, or 30 minutes to devote, to really sit down and go through it, made it a little hard. And I guess where, if I'd had it on my phone, I could've come home and done it. But that was the only negative thing that I could say.” Similarly, a few participants noted the limited Internet access in the region could be a barrier to others (*e.g.*, “For me, you know, I have access to Internet and stuff. [But] a lot of people don't or if I was one that didn't have Internet or had to go to a library to be able to do it, you know it would be hard”).

## Discussion

The National Institutes of Health (NIH) has recognized that sleep and the health of women is an important research focus.^6^ Roughly a third of women report insomnia, and the percentage impacted by insomnia increases with age.^5^ Because insomnia is associated with a host of negative health outcomes, including obesity, cardiovascular disease, and diabetes, increased interventional and translational approaches are particularly important for women who are members of health disparity populations.^5,6^

Appalachian women represent a unique intervention target due to a confluence of factors. They are disproportionately affected by insufficient sleep^12^ and their biopsychosocial characteristics (*e.g.*, female sex, older age, high depression rates) intersect with cultural (*e.g.*, strong self-reliance norms) and regional (*e.g.*, limited transportation) factors that complicate their treatment trajectories.^10^ Appalachian women additionally face regional health care professional shortages and prevalent prescription drug abuse.^10,15^ A technologically facilitated nonpharmacological insomnia intervention such as SHUTi may be ideally suited to improve sleep in this population. Potential barriers, including documented suspicion of technology, necessitated a pilot test of SHUTi's feasibility and accessibility.^12,16^

Although impeded Internet access and technology use overall may limit access to this web-based program for all rural Appalachian women, our participants adhered to and expressed enthusiasm for SHUTi. The 82.6% participant retention rate is consistent with previous SHUTi interventions.^17,20^ Participants generally followed the SHUTi protocol and most rated their experience as satisfactory and helpful. Participants described SHUTi as a good, helpful, educational, and interesting program. In line with previous research on Appalachians' self-reliance norms,^14,16^ our participants liked the flexible independent nature of SHUTi. Moreover, given significant transportation limitations, geographic isolation, and challenging road conditions, participants endorsed a program that can be used at a location and time convenient to them.

Suggestions for improvement centered on extending the sleep diary window and potentially reducing the program's duration. Although all of our participants had access to the Internet either at home or at work, a few participants noted that access could be a barrier for others in the region. Overall, results from our pilot study suggest that SHUTi was feasible and acceptable for middle-aged Appalachian women. These results complement our findings that SHUTi is effective in significantly improving insomnia severity, sleep quality, perceived stress, depression symptoms, and reducing sleep medication use in this population.^23^

Our findings also directly address the call of the NIH's Conference on Sleep and the Health of Women to implement and evaluate technologically facilitated interventions in hard-to-reach health disparity populations.^8^ Technology has demonstrated benefit in compensating for the lack of specialists, inaccessible transportation, and overall community resource shortages in rural communities.^32,33^ In addition, improved access to health records through online patient portals, health education search engines, and telemedicine, among other technologies, have been instrumental in patient empowerment, reductions in travel time to, and wait times for, appointments, and continuity of care.^34^ Although lack of access to computers and smartphones as well as inconsistent Internet access and limited mobile reception are potential barriers to using Internet-based interventions in rural areas,^35^ our findings align with other work suggesting that rural residents, including those from Central Appalachia, increasingly use and are favorably oriented toward personal technology.^36,37^

This study had multiple limitations, including lack of random assignment, postintervention follow-up, and a control group. As we did not have access to participant medical records, exclusion criteria and outcomes were based on self-report. When compared with the average female Kentucky resident, a higher percentage of study participants had private insurance and greater educational attainment. In addition, intervention completers were more likely to have private insurance compared with noncompleters. It is possible that study completers had access to more financial and health care resources, including insurance. For example, those with insurance also may have resource advantages such as more access to the Internet (*i.e.*, at home and work instead of work only) or better awareness of community resources. Nevertheless, all Appalachian women are considered members of a health disparity population, due to their geographic location, sparsely resourced rural environment, limited access to health care, scarce community resources, and increased risk of morbidity and mortality.^9,10^

Although our sample was largely representative of the target population (Appalachian women ages 45+ years with insomnia), its small size, racial/ethnic homogeneity, and the inherent selection bias from voluntary participation limit generalizability. Future research should incorporate a randomized case–control design, within a larger and more diverse sample.

## Conclusions

Insomnia, a disorder most prevalent in women ages 45+ years, is linked to numerous negative health outcomes.^1–3,13^ Our study is the first to quantitatively and qualitatively assess the feasibility and acceptability of an Internet-based insomnia intervention in the health disparity population of Appalachian women. We conclude that CBT-I interventions available through an Internet connection and/or a mobile phone, although currently underutilized, may prove essential to reaching vulnerable rural women experiencing insomnia.

## Abbreviations Used

CBT-I: cognitive behavioral therapy for insomnia
CI: confidence interval
NIH: National Institutes of Health
PI: principal investigator
SD: standard deviation
SHUTi: Sleep Healthy Using the Internet
